# Changes of polyphenols and antioxidants of arabica coffee varieties during roasting

**DOI:** 10.3389/fnut.2023.1078701

**Published:** 2023-01-26

**Authors:** Marilu Mestanza, Pati Llanina Mori-Culqui, Segundo G. Chavez

**Affiliations:** Instituto de Investigación para el Desarrollo Sustentable de Ceja de Selva, Universidad Nacional Toribio Rodríguez de Mendoza de Amazonas, Chachapoyas, Peru

**Keywords:** Bourbon, Catimor, Caturra, bioactive compound, green grain

## Abstract

Coffee is the most consumed beverage in the world after water. Multiple benefits are attributed to it in human health due to the presence of antioxidant compounds, whose content depends, among other factors, on the processing conditions of the coffee bean. The objective of this study was to determine the kinetics of polyphenols and antioxidants during the roasting of three varieties of arabica coffee. For this, we worked with varieties of coffee, Catimor, Caturra, and Bourbon, from the province of La Convencion, Cuzco, Peru. The samples were roasted in an automatic induction roaster, and 12 samples were taken during roasting (at 0, 1, 3, 5, 7, 9, 11, 13, 15, 17, 19, and 21 min of roasting) in triplicate. For green coffee beans, titratable acidity, total soluble solids, moisture and apparent density were determined. The change in polyphenol content was determined using the Folin-Ciocalteu method, and antioxidant activity was determined using the 2,2-diphenyl-1-picrylhydrazyl (DPPH) and 2,2-azino-bis- (3-ethyl-benzothiazoline-6-sulfonic acid) (ABTS^+^) free radical capture technique during roasting. Polyphenol and antioxidant contents increased until minute 5 of roasting and then decreased until minute 20, and in some cases, there were slight increases in the last minute. The model that best described the changes in these bioactive compounds was the cubic model (*R*^2^ 0.634 and 0.921), and the best fits were found for the Bourbon variety, whose green grain had more homogeneous characteristics. The changes in the relative abundances of nine phenolic compounds were determined using high-performance liquid chromatography (HPLC). In conclusion, roasting modifies phenolic compounds and antioxidants differently in the coffee varieties studied. The content of some phenols increases, and in other cases, it decreases as the roasting time increases. The roasting process negatively affects the bioactive compounds and increases the fracturability of Arabica coffee beans, elements that should be taken into account at the moment of developing roasting models in the industry.

## 1. Introduction

After water, coffee is the most widely consumed beverage in the world and probably the most economically relevant agricultural product because since it is the most important commodity after oil (1).

The consumption of coffee has been associated with controversial nutritional approaches; however, recent research has shown a relationship between the habitual consumption of coffee beverages and a reduced risk of chronic diseases, including cancer (2).

Caffeine is the main compound in coffee, both because it is the best known and because of the properties it confers on the beverage. As well as being a potent central nervous system stimulant, improving mood, its consumption is associated with a reduction in the risk of suffering from Parkinson’s disease and Alzheimer’s disease and with hepatoprotective effects (3). Additionally, coffee is a source of large amounts of minerals and bioactive compounds (4), such as chlorogenic acid, caffeic acid and other phenolic compounds (5, 6), which are responsible for its high antioxidant power.

The roasting process causes physical, chemical and biological changes in coffee beans (7, 8). Previous experiments have shown that the change in the antioxidant activity of the beverage is closely associated with changes in bioactive compounds such as chlorogenic acid (9–11).

The total phenolic and flavonoid content and antioxidant activity of coffee beverages decrease with increasing roasting degree (12, 13). Similarly, although caffeine has a high melting point, the high temperatures used in the coffee roasting process can affect its content beverages (14). Therefore, the high temperatures to which coffee beans are subjected to in order to produce volatile aromatic compounds, as in other foods, degrade compounds of high nutritional value, such as antioxidants (15, 16). By degrading these compounds, the antioxidant activity of coffee beverages is considerably reduced, and a relative increase in caffeine content can be observed due to its high thermal stability (6, 17).

Although the phenol content in robusta coffee has been shown to be higher than that in arabica coffee, both are proportionally affected by roasting processes (18). In addition, the antioxidant content in the final beverage depends on the species, variety, processing technique and preparation (19).

This research work deals in depth with the changes of phenolic compounds during coffee roasting. The kinetic models of phenolic composition and antioxidant activity in the coffee beverage are specified. In addition, the changes in coffee bean fracturability during the roasting process, a very important parameter to standardize roasting models, are exposed.

It has been shown that the roasting of coffee beans degrades compounds responsible for the antioxidant activity of coffee beverages, it unknown how their quantities change. Therefore, the aim of this study was to investigate the changes in polyphenol content and antioxidant activity during the roasting of three varieties of arabica coffee grown in Peru.

## 2. Materials and methods

### 2.1. Material

In this research, green arabica coffee beans of Bourbon, Caturra, and Catimor varieties from the province of La Convencion, Cuzco, Peru, were used. Green coffee beans with sensory scores greater than score 88 in SCA/CoE cupping (20) was acquired, and all subsequent processing was performed in the Coffee Processing and Quality Control Laboratory of Universidad Nacional Toribio Rodríguez de Mendoza de Amazonas.

### 2.2. Experimental procedure

**FIGURE 1:**
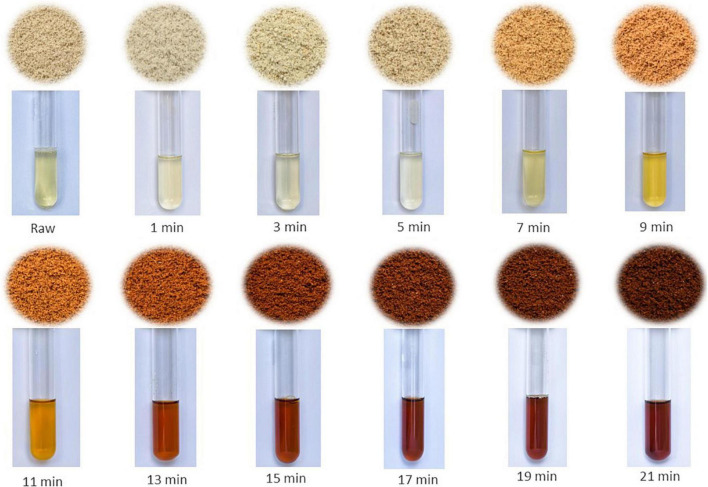
Ground coffee and its respective extracts obtained during the roasting process.

The coffee was selected using 17 mesh and then roasted for 21 min in a Probat electric sample roaster (Germany) with a starting temperature of 170°C, 60% heating power and 200 g load per batch. A sample or approximately 2 to 3 g was taken every 2 min (at 0, 1, 3, 5, 7, 9, 11, 13, 15, 17, 19, and 21 min of roasting) (Figure 1) of three arabica coffee varieties (Bourbon, Caturra, and Catimor); then 3A × 12B bifactorial experiment was run. Each treatment was performed in triplicate, and the samples were stored in polyethylene flasks with a lid under refrigeration (4–8°C) until further treatment.

### 2.3. Infusions

The coffee samples taken at different roasting times were ground in a bean grinder (Bosch, Germany). Then, infusions were obtained by adding hot 180 mL of water (90°C) to beakers with 10 g of ground coffee. Infusions lasted 5 min, after which the sample was filtered through filter paper (Whatman no. 40). The extracts were stored under refrigeration (4°C) until subsequent analysis.

### 2.4. Determination of antioxidant activity

Antioxidant activity was determined using two techniques. (1) The free radical 2,2-diphenyl-1-picrylhydrazyl (DPPH) was assessed using the technique developed by Brand-Williams et al. (21) and adapted by Çelik and Gökmen (22) for the determination of antioxidant activity in coffee. A methanolic solution of DPPH (20 mg/L) was prepared to obtain an absorbance of approximately 0.45. DPPH solution (3.9 ml) was used and placed in glass cuvettes to measure the initial absorbance of DPPH (A0) at a wavelength of 516 nm. Then, 100 μL of sample extract was pipetted and placed in the cuvette containing the DPPH solution and stirred. The cuvettes were left in the dark for 10 min, and then, final absorbance (TA) was measured.

The decrease in absorbance of the resulting solution was measured spectrophotometrically at 516 nm (UV/VIS spectrometer). All experiments were performed in triplicate, and the mean values were reported. The scavenging capacity was calculated using the following equation and expressed as the inhibition of DPPH:


$$\%\text{ inhibition of DPPH}=\frac{\left(\text{A}0-\text{AS})-(\text{TA}-\text{AS}\right)}{\left(\text{A}0-\text{AS}\right)}X100$$

where A0 is the absorbance of the DPPH solution, AS is the absorbance of methanol, and TA is the absorbance of the sample.

(2) The uptake of the 2,2-azino-bis- (3-ethyl-benzothiazoline-6-sulfonic acid (ABTS^+^) radical was assessed as described by Del Castillo et al. (23). The Trolox standard was used for all, and the values were expressed in mmol Trolox equivalent/liter of infusion. To generate the ABTS^+^ cation, 19.2 mg of 2,2′-azino-bis (3-ethylbenzothiazoline-6-sulfonic acid) was weighed and dissolved in 5 mL of distilled water to obtain a concentration of 7 mM. Then, 88 μL of 140 mM potassium persulfate was added. The resulting solution was homogenized and incubated at room temperature (25°C ± 1) in the dark for 16 h. Once the ABTS^+^ radical was generated, the solution was adjusted with methanol until obtaining an absorbance of 0.7 ± 0.1 at 754 nm. For the analysis of samples, 3.9 mL of ABTS^+^ solution was used, 100 μL of the aqueous coffee extract was added, and the mixture was stirred vigorously. The sample was immediately read in a UV/VIS spectrophotometer at 754 nm. A calibration curve was constructed with Trolox (concentration range of 0 to 1.0 mM).

### 2.5. Total phenol content

For each of the extracts and their respective replicates, the total polyphenol content was determined using the Folin-Ciocalteu technique, following the procedure described by Çelik and Gökmen (22). Gallic acid was used as the standard; 10 mg of gallic acid was weighed and diluted with ultrapure water to a volume of 100 mL to prepare a stock solution. This solution was used to prepare the calibration curve (concentration range of 0–16 mg/L). Extracts (50 μL) diluted in 950 μL of ultrapure water were pipetted into test tubes, and then 2.5 mL of Folin-Ciocalteu reagent diluted in distilled water (1:10 v/v) and 2 ml of Na_2_CO_3_ (20% aqueous solution) were added. The test tubes were placed in an oven at 50°C for 5 min to allow the development of a blue complex. All samples were prepared in triplicate. Absorbance was measured in a UV/VIS spectrophotometer at a wavelength of 765 nm.

### 2.6. Chromatographic conditions for the determination of phenolic compounds in coffee beverages

Phenolic compounds were separated using high-performance liquid chromatography (HPLC) following the method described by Brunetto et al. (24) in a Hitachi-Chromaster chromatograph (Tokyo, Japan; LC-20AD) equipped with an SIL-20A/HT autoinjector, a CBM-20A communication module and an SPD-M20A diode array detector (DAD). UV detection was recorded at 278 nm. Separation was performed on a Supelco-LiChrospher RC C-18 column (5 μm; 25 cm × 4.6 mm). A mixture of methanol/water (30/70 v/v) was used as the mobile phase in isocratic mode at a flow rate of 1.0 mL/min. Phenolic compounds were identified only by retention time because there were no standards for identification by name.

### 2.7. UV-vis spectral scanning

For the spectral analysis, the methodology described by Tian et al. (25) was used with some modifications. Coffee extracts were diluted 100-fold with distilled water. The diluted sample (3 mL) was added to a quartz cuvette and inserted into the reading cell of the UV-vis spectrophotometer (T 9200 PEAK, USA). Distilled water (3 mL) was used as calibration reference. The reading range was between 190 and 400 nm in triplicate.

### 2.8. Fracturability

A CTX texture analyzer (AMETEK Brookfield, USA) with TexturePro 1.0.19 software, equipped with a 100 kg load cell, was used. The test was performed with a cylindrical probe of 6 mm diameter and 35 mm length, a test speed of 3 mm/s and a depth of 0.25 mm. The pre-test velocity was 5 mm/s, data acquisition of one point per second and 1 N as activation load. Fracturability was calculated and values in N are reported.

### 2.9. Data analysis

To compare between treatments we used analysis of variance and Tukey’s test of comparisons to determine the significance between groups (sig. = 0.05), using SPSS V.26 statistical software. The results presented in tables contain mean values and standard deviations and figures were produced to demonstrate trend and kinetic models.

## 3. Results

Table 1 provides the characteristics of the coffee used in the experiments. Except for the soluble solids content, the samples used were different. Green coffee of the Bourbon variety had a higher acidity content than did the other two varieties used. The green coffee variety Caturra had the highest moisture content (11.38%) and the highest apparent density (778 g/L).

**TABLE 1:** Physicochemical characteristics of green gold coffee of three Arabica varieties.

Variety	Acidity (%)*	SST (%)*	Humidity (%)*	Apparent density (g/L)*
Bourbon	1.50 ± 0.00a	2.97 ± 0.05a	10.41 ± 0.02c	772 ± 1.01b
Caturra	1.13 ± 0.00b	2.75 ± 0.09a	11.38 ± 0.03a	778 ± 1.91a
Catimor	1.13 ± 0.22ab	2.90 ± 0.10a	10.98 ± 0.08b	759 ± 2.67c

*Averages and standard deviations (*n* = 3). Different letters indicate significant differences among groups (*p* < 0.05), Tukey’s test.

Green Bourbon coffee beans had a higher phenolic content than did the beans of the Caturra and Catimor varieties (66.59 compared to 53.31 and 56.74 mg AGE/g, respectively). As seen in Table 2, when the roasting process began, the phenolic content increased, peaking at minute 3 for the Bourbon variety (80.35 mg AGE/g) and minute 9 for the Caturra and Catimor varieties (69.46 and 67.55 mg AGE/g, respectively). After that time, the phenolic content decreased proportionally below the initial phenol content.

**TABLE 2:** Effect of roasting on the phenol content and antioxidant capacity of three coffee varieties (Bourbon, Caturra, and Catimor).

Variety	Roasting time (min)	PFT (mg AGE/g)*	ABTS (mmol TE/L)*	DPPH (mmol TE/L)*
Bourbon	0	66.59 ± 3.66abc	11.23 ± 0.12b	19.50 ± 1.57f
	1	71.60 ± 4.05abc	11.31 ± 0.16b	23.97 ± 0.42cde
	3	80.35 ± 11.44a	11.68 ± 0.04a	31.17 ± 0.81ab
	5	75.15 ± 14.11ab	11.70 ± 0.02a	34.11 ± 1.17a
	7	75.74 ± 9.38ab	11.66 ± 0.02a	32.53 ± 0.63a
	9	79.38 ± 3.41a	11.61 ± 0.01a	27.96 ± 1.24bc
	11	75.53 ± 6.09ab	11.59 ± 0.02a	27.08 ± 1.09bcd
	13	68.48 ± 2.75abc	11.54 ± 0.02a	23.42 ± 1.67def
	15	62.01 ± 7.55abc	11.52 ± 0.05a	21.14 ± 1.31fg
	17	60.57 ± 4.23abc	11.55 ± 0.06a	19.71 ± 0.95fg
	19	55.66 ± 5.85bc	11.54 ± 0.07a	14.33 ± 2.31g
	21	50.45 ± 2.77c	11.58 ± 0.04a	15.06 ± 2.85g
Caturra	0	53.31 ± 4.35bc	11.36 ± 0.11b	30.22 ± 1.95de
	1	55.20 ± 4.08abc	11.42 ± 0.07bc	32.38 ± 2.95cde
	3	57.06 ± 3.71abc	11.75 ± 0.06a	41.12 ± 0.55a
	5	69.94 ± 13.47ab	11.77 ± 0.05a	42.17 ± 0.87a
	7	75.07 ± 10.55a	11.71 ± 0.06a	41.66 ± 1.64a
	9	69.46 ± 5.66ab	11.69 ± 0.06a	38.96 ± 1.91ab
	11	63.97 ± 10.21abc	11.65 ± 0.04a	37.40 ± 2.28abc
	13	55.84 ± 6.93abc	11.64 ± 0.08a	34.15 ± 2.07bcd
	15	53.17 ± 5.33bc	11.63 ± 0.09ab	31.96 ± 2.48cde
	17	50.62 ± 3.66bc	11.66 ± 0.04a	31.81 ± 1.75cde
	19	46.87 ± 4.00c	11.62 ± 0.07ab	28.60 ± 1.32de
	21	45.26 ± 2.19c	11.61 ± 0.09ab	27.34 ± 1.69e
Catimor	0	56.74 ± 1.58bcd	11.36 ± 0.11b	31.63 ± 2.63ef
	1	59.45 ± 4.38a-d	11.42 ± 0.12b	34.90 ± 2.17c-f
	3	64.25 ± 2.59ab	11.70 ± 0.01a	41.93 ± 2.40ab
	5	67.09 ± 2.74a	11.74 ± 0.00a	43.37 ± 1.92a
	7	62.24 ± 2.47abc	11.73 ± 0.04a	44.59 ± 1.05a
	9	67.55 ± 1.20a	11.69 ± 0.02a	42.48 ± 2.56ab
	11	57.92 ± 2.55a-d	11.68 ± 0.02a	41.10 ± 1.94abc
	13	52.64 ± 6.57cde	11.62 ± 0.08a	39.19 ± 1.64a-d
	15	50.69 ± 4.32def	11.64 ± 0.06a	35.92 ± 1.96b-e
	17	46.01 ± 3.48ef	11.65 ± 0.08a	34.18 ± 2.73def
	19	43.41 ± 1.20ef	11.64 ± 0.05a	32.58 ± 2.78def
	21	40.46 ± 4.53f	11.63 ± 0.05a	28.26 ± 2.95f

*Averages and standard deviations (*n* = 3). Different letters indicate statistically different groups with 0.05 significance according to Tukey’s test, by coffee variety.

Regarding the antioxidant capacity of the infusions, the ABTS technique seemed to be less sensitive; however, the trend was similar to that for total phenol content (the initial values increased with time and then decreased) for the three varieties of coffee studied. The DPPH radical capture technique was apparently more sensitive than the ABTS technique for determining the antioxidant capacity of the infusions. Unlike the total phenol content, green coffee of the Bourbon variety had the lowest antioxidant capacity (19.50 vs. 30.22 and 31.66 mmol TE/L, respectively); however, the evolution over time was correlated in such a way that the maximum values were obtained in minutes 5, 9, and 7 for Bourbon, Caturra and Catimor, respectively, and then at minute 21 decreased to values lower than the initial values.

### 3.1. Changes in total polyphenols during coffee roasting

**FIGURE 2:**
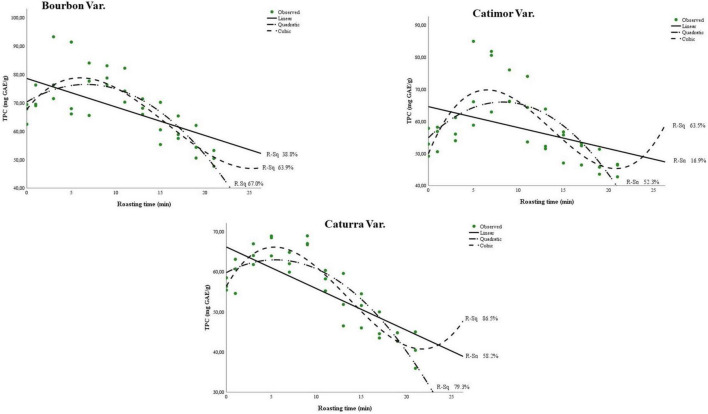
Models of polyphenol degradation during roasting of three coffee varieties (Bourbon, Catimor, and Caturra).

Figure 2 and Table 3 show the curves for the change in total polyphenol content during the roasting of the three coffee varieties. Although the three models, i.e., linear, quadratic and cubic, are significant (sig. < 0.05), the changes in phenol content during roasting better fit the cubic model (*R*^2^ > 0.63). More precise models were obtained for the Catimor variety (*R*^2^ of 0.58; 0.79 and 0.86 for the linear, quadratic, and cubic models, respectively) than for the Bourbon and Caturra varieties. In all treatments, a decrease in phenolic content is observed during roasting of the coffee bean (Figure 2).

**TABLE 3:** Summary of the models and estimated parameters of polyphenol degradation during the roasting of three coffee varieties.

Variety	Model	*R* ^2^	Sig.*
Bourbon	Linear	0.388	0.000
	Quadratic	0.639	0.000
	Cubic	0.670	0.000
Caturra	Linear	0.169	0.013
	Quadratic	0.523	0.000
	Cubic	0.635	0.000
Catimor	Linear	0.582	0.000
	Quadratic	0.793	0.000
	Cubic	0.865	0.000

*Statistical significance of the regression model.

### 3.2. Change in coffee antioxidants during roasting

**FIGURE 3:**
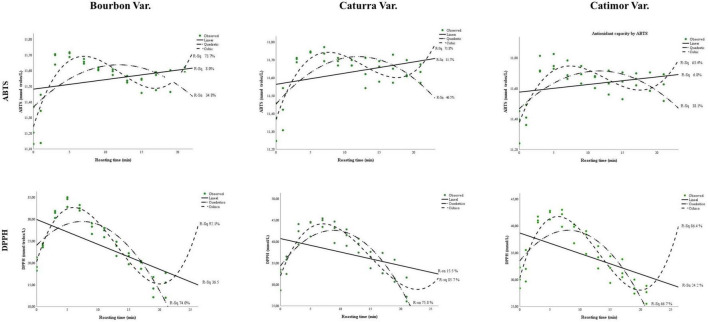
Changes in antioxidants in three varieties of coffee (Bourbon, Caturra, and Catimor) during roasting.

Figure 3 and Table 4 show the values and evolution of the antioxidant capacity of coffee during roasting.

**TABLE 4:** Summary of kinetic models of antioxidant activity by capture of the ABTS radical.

Variety	Model	*R* ^2^		Sig.*	
		ABTS	DPPH	ABTS	DPPH
Bourbon	Linear	0.080	0.365	0.095	0.000
	Quadratic	0.348	0.740	0.001	0.000
	Cubic	0.737	0.921	0.000	0.000
Caturra	Linear	0.068	0.242	0.124	0.002
	Quadratic	0.381	0.667	0.000	0.000
	Cubic	0.634	0.864	0.000	0.000
Catimor	Linear	0.117	0.155	0.041	0.018
	Quadratic	0.465	0.758	0.000	0.000
	Cubic	0.718	0.857	0.000	0.000

*Statistical significance of the regression model.

As seen in Figure 3, compared with the ABTS method, the DPPH free radical capture technique showed greater changes in the antioxidant capacity of coffee beans during roasting. Both techniques allowed us to observe that immediately after starting the roasting process, the antioxidant capacity increased up to a certain time (5 to 9 min, as seen in Table 2). Then, the capacity decreased, and at the end of roasting, a slight increase was observed (Figure 3).

With the DPPH radical capture technique, models with greater fit were obtained than with the ABTS method, with *R*^2^ values greater than 0.85 for the three coffee varieties studied.

**FIGURE 4:**
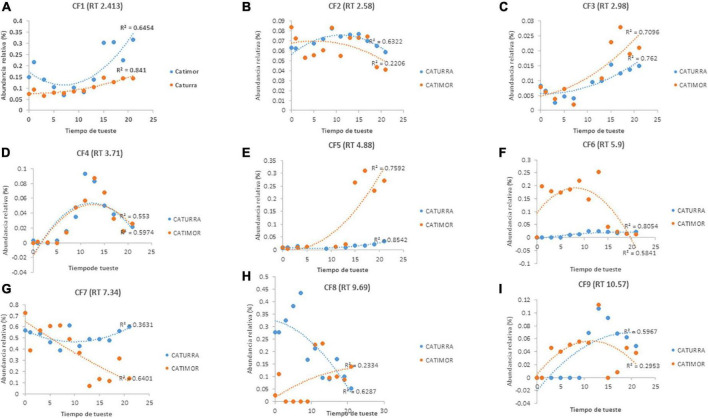
Evolution of phenolic compounds during the roasting of two varieties of Arabica coffee. **(A–I)** Phenolic compounds identified according to retention time. PC, phenolic compound; RT, retention time.

Figure 4 shows the changes of nine phenolic compounds in coffee during roasting. Four of them (Figures 4B, D, F, I) increased their content up to the 10th minute of roasting and then decreased, and three other compounds (Figures 4A, C, E) increased until the end of roasting. Figures 4G, H show that there are phenolic compounds that are affected from the beginning of the roasting process.

### 3.3. Change in phenolic composition of arabica coffee during roasting

**FIGURE 5:**
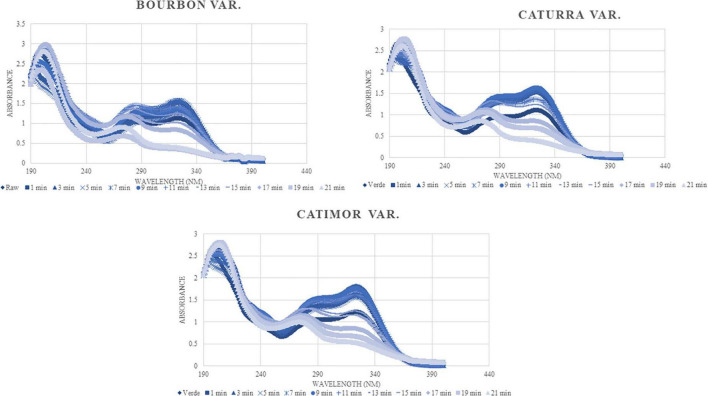
Change in the phenolic composition of coffee during roasting (UV-Vis spectrum).

As can be seen in Figure 5, and as expected, all the spectra have the same trend; however, there are differences in the content of compounds identified, basically in the detection spectrum of phenolic compounds (240–340). The phenolic content of the raw samples increases until minute 9 of roasting and then decreases as a result of the process.

### 3.4. Fracturability of arabica coffee beans during roasting

**FIGURE 6:**
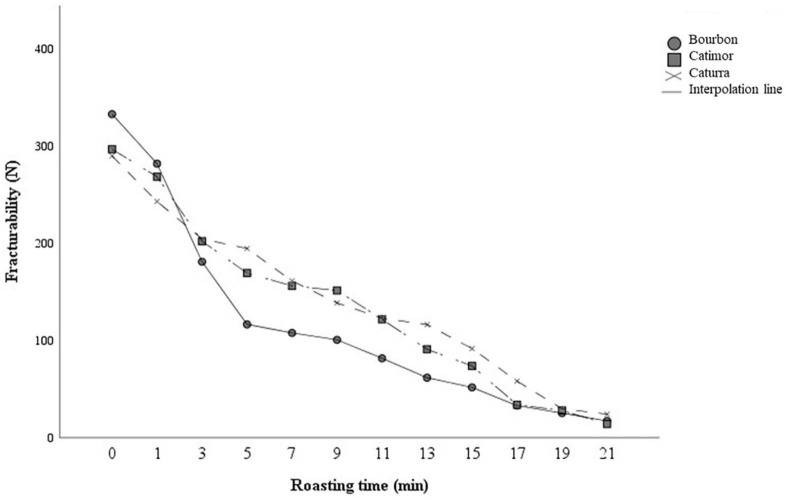
Changes in the fracturability of coffee beans during the roasting process. Curves represent averages (*n* = 3).

Figure 6 shows that as the degree of roasting increases, the beans are easier to break. During the first 5 min of roasting, there is a reduction in hardness that is maintained, but moderately until the end of the roasting process. In addition, there are significant differences between varieties (*p* < 0.05). Bourbon coffee requires greater force to fracture the beans during the first minutes, but from minute three onward it presents greater bean fracturability compared to Catimor and Caturra.

## 4. Discussion

Coffee roasting times usually range from 8 to 12 min, depending on the degree of roasting required (26, 27). In this research, the roasting time was extended to 21 min to determine changes in bioactive compounds.

Although the variety of coffee is a determining factor with regard to its chemical characteristics (28), the post-harvest process conditions should also be taken into account. The unroasted samples of the Bourbon variety had higher acidity (1.50%) and lower moisture content (10.41%) and density (772 g/L), in contrast to the two others varieties studied, which could influence the results (29). In fact, for this variety, the kinetic models of antioxidant activity had a greater fit (*R*^2^ = 0.92 for the cubic model), and in terms of total phenol content, the best fit was obtained for the Catimor variety.

As in most foods (30), phenolic compounds are mainly responsible for the antioxidant activity in coffee. Kinetic models of the phenolic content of coffee during roasting are different from models describing changes in antioxidant activity. This is because although phenolic compounds are the main antioxidants in coffee, there are other compounds such as those derived from the Maillard reaction that have the capacity to capture free radicals (31). The phenol content in the coffee samples evaluated was higher than those reported by Bobková et al. (32), who obtained values lower than 74 mg AGE/g; in this study, for the Bourbon variety, 80 mg AGE/g of sample was obtained at 5 min of roasting, which could be considered, given the relevance of phenolic compounds (33), for the production of coffee beverages with high bioactive potential.

As reported in other studies (34), roasting favors an increase in phenol content in coffee beans. However, when the time is prolonged (after 5 or 9 min depending on the variety of coffee), a decrease in these compounds is observed, which could be due to the degradation of chlorogenic acid, one of the most important phenols in coffee (11); caffeic acid, on the other hand, is not affected by temperatures below 200°C.

Previous studies have found that green coffee beans have higher antioxidant activity than roasted beans (11, 34). This study clarifies the phenomenon, revealing that in the first minutes there is an increase in antioxidant capacity; however, if the time is prolonged (more than 9 min), the antioxidant capacity decreases to values lower than the initial values.

The ABTS and DPPH radicals allow slightly different approaches to measure antioxidant activity. DPPH reacts with polyphenols but not with phenolic acids (35). Therefore, the sharp decrease in the kinetic models of antioxidant activity could be due to the degradation of phenolic compounds and the formation of phenolic acids, which are captured by ABTS, whose curves reduce less as the roasting time increases (Figure 2).

The initial increase in phenolic compounds and antioxidant capacity of coffee beans during roasting occurs because temperature facilitates the release and generation of antioxidant compounds (36). However, higher temperatures and roasting times may not only degrade bioactive compounds but also generate undesirable compounds in beverages, such as acrylamides (37). This allows us to highlight the importance of the development of roasting models that seek to obtain coffee beverages rich in bioactive compounds but low in compounds harmful to health (38).

Although unroasted green coffee with low roast degrees has a higher phenol content and greater bioactive properties (39), one of the purposes of roasting is to generate aromatic compounds that confer great sensory acceptance. Therefore, roasting modalities should seek to obtain beverages with high acceptance and with the greatest amount of bioactive compounds (34), as assessed *via* sensory analysis techniques with panelists or instrumentals (40).

Nine phenolic compounds were detected; for seven of these compounds, their concentration tended to increase (Figures 4A–F, I), although the concentration of four compounds (Figures 4B, D, F, I) began to decrease during the roasting process. The behavior of these compounds is similar to that reported in other studies of the phenol profile of green and roasted coffee, reporting that the concentrations of gallic acid and caffeic acid increase due to hydrolysis of chlorogenic acids (9); in this work it was shown that when the roasting time is prolonged (more than 10 min), these compounds are affected and their content is reduced. In contrast, the concentrations of two phenolic compounds (Figures 4G, H) decreased from the start of roasting, similar to what occurs with chlorogenic acid, the main phenolic compound in coffee, and other phenolic compounds with antioxidant potential (8, 9, 11, 41).

As demonstrated in previous studies, the UV-Vis spectrum at certain wavelengths, allows observing changes in the content of phenolic compounds (25, 42, 43). Most phenolic compounds are detected between 200 and 340 nm (44). Therefore, changes in caffeine and chlorogenic acid content can be observed in that range of analysis, the content of which decreases as the degree of roasting of coffee increases (45).

During the roasting process coffee beans undergo physical, chemical, structural and sensory changes (46). The hardness of coffee beans is affected by roasting conditions, in effect the beans lose strength and hardness, and become increasingly brittle (47). This effect of roasting has been observed in the three varieties of coffee studied in this research. Coffee beans of the Bourbon variety reduce their fracturability faster than the Caturra and Catimor varieties during roasting, which would influence the grinding process. The differences between varieties could be attributed to the moisture content, anatomy and density of the beans, since these factors influence the increase in volume, porosity and pyrolysis reaction during roasting (47, 48).

## 5. Conclusion

Both the phenolic content and the antioxidant capacity of coffee beans are affected by roasting. Both increase up to minutes 5 and 9 depending on the variety and then decrease to concentrations below the initial value when roasting lasts longer than necessary. Changes in antioxidant compounds during roasting depend on the variety and the physicochemical characteristics of the coffee bean. The cubic mathematical model is the one that best fits the changes in phenolic and antioxidant compounds. The hardness of the coffee bean also decreases with roasting. Finally, it is confirmed that the roasting process negatively affects bioactive compounds and increases the fracturability of coffee beans, elements that should be taken into account when developing roasting profiles in the industry.

## Data availability statement

The original contributions presented in this study are included in the article/supplementary material, further inquiries can be directed to the corresponding author.

## Author contributions

MM and SC: conceptualization. MM and PM-C: methodology. SC, MM, and PM-C: formal analysis, research and writing—preparing the original draft, and writing—revision and editing. SC: funding acquisition. All authors contributed to the article and approved the submitted version.
